# Inspiration and curiosity: encouraging careers with postgraduate dental research

**DOI:** 10.1038/s41415-024-7845-2

**Published:** 2024-12-13

**Authors:** Martin. E. J. Curzon

**Affiliations:** Emeritus Professor of Child Dental Health, University of Leeds, Leeds, UK

## Abstract

The role of teachers and mentors is crucial in developing young members of the dental profession to consider an academic career. In this paper, the experience of the author, as a young postgraduate in paediatric dentistry at the Eastman Dental Center (Rochester, New York), is described when his curiosity was aroused concerning the possible causative or preventive effects of trace elements on tooth enamel. That curiosity was encouraged by Dr Basil Bibby, Director of the Eastman Dental Center, who inspired many students to conduct a research project. Further guidance instigated a change in the author's life plans, leading ultimately to a research and academic career.

## Key points

- Curiosity in dental students should always be encouraged.
- Senior staff should inspire students by their example.
- Through mentoring students can be advised and guided into research careers.

## Introduction

When setting out on a career in dentistry, most British students' prime aim is to qualify but without any set ideas as to how to practise dentistry after qualifying. A few dental students have ambitions to become a consultant, the majority will want to open their own practice and a small number want to enter the community dental services. However, there are sometimes a few students in any graduating class that decide to travel before settling down to general practice. An aspect of travelling and working in different institutions is to encounter and possibly be influenced by elder leaders and teachers for alternative career possibilities.

## The student

Qualifying at University College Hospital School of Dentistry in 1964, I decided to travel before settling down and was appointed as an itinerant paedodontic extern travelling the backwoods of British Columbia. The charge was to provide comprehensive dental care for children. As a result of that experience, I decided to specialise in paediatric dentistry and with that in mind, I gained a place on the two-year programme at the Eastman Dental Center (EDC). Accordingly, I arrived in Rochester (New York) in September in 1966 to join a group of some 24 postgraduate students to study various clinical aspects of dentistry, one group being in paediatric dentistry. Interestingly, the new students came from many countries, including the British Isles, Canada, France, Sweden, Ireland, Egypt and Denmark, although primarily from the USA. In the first two terms, all students attended all lectures and seminars every morning at 8.30 am. One such course was given by the Director of the EDC, Dr Basil Bibby, an internationally renowned teacher and researcher whose own interests focused on oral bacteriology, dental caries and prevention. I had not heard of Dr Bibby but was soon to discover his widespread reputation.

## Basil Bibby

**Fig. 1:**
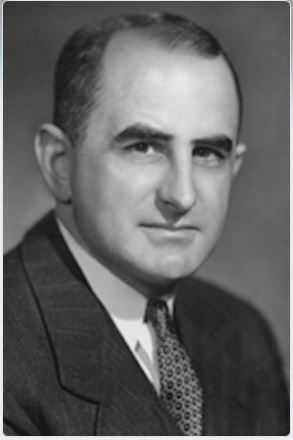
Dr Basil Bibby. Image courtesy of Eastman Dental Center papers, Miner Library, University of Rochester Medical Center

Born in Waipawa, New Zealand in 1904, Basil Bibby graduated in dentistry at the University of Otago in 1927. In his younger years, he had been a junior rugby All Black and played for Otago University. After qualifying, he was inspired to start a research project under the influence of the dean of Otago, Henry Pickerill, publishing his first paper in 1931 titled *‘*A study of a pigmented dental plaque'.^1^ Encouraged to travel by Pickerill and to consider a research career in dentistry, Basil was awarded a Rockefeller Fellowship to study at the University of Rochester (New York) and commenced his PhD under the guidance of Nobel Laureate Professor George Whipple. Basil's main interest was the identification of oral bacteria and dental caries. Completing his doctorate in 1935,^2^ Basil taught at Rochester before moving to Tufts Dental School as professor of bacteriology and dean in 1944. However, soon afterwards, in 1947, Dr Bibby was appointed director of the EDC, where he developed and expanded his ideas on postgraduate students' studies and research. For his time, he was the most renown dental researcher with a world-wide reputation and as such was an authority on all aspects of dental caries (Fig. 1).

Many young dentists came to study under Bibby and went on to pursue successful careers as researchers and teachers. The EDC became highly regarded as an important centre of postgraduate studies. Many students were guided by Basil Bibby but there were others by the eminent members of staff that Basil had recruited.

## Curiosity

Although Basil Bibby was the director of the EDC, he also played a very active role in postgraduate teaching. The course he presented was titled simply ‘Dental caries' and was held early every Tuesday morning over many months. Each lecture covered one aspect of the disease, including historical studies, epidemiology, factors affecting susceptibility, prevention and what approaches could be used to increase resistance.

The lecture titled ‘Environmental aspects of dental caries' concerned food, diet and nutrition and discussed the relationship of where foods were grown and hence how the expression of dental decay might be related to composition of food, water supplies, soils and geology. As examples, Dr Bibby noted evidence that caries was related to hard or soft water supplies and to the soils or rock strata through which the water flowed. But there were instances when the water might take up toxic elements, detrimental to tooth composition, and he gave one example that had been reported by geologists of poisoned agricultural soils affected by the heavy metals zinc (Zn), lead (Pb) and cadmium (Cd). The poisoned soils in question were discovered in an area of upstate New York in Orleans County, only a few miles due west of Rochester. I found that lecture most intriguing and later that same day dropped by the director's office to ask Dr Bibby for more details. As usual, Basil was dressed in his old brown laboratory coat sitting at his desk. Calling me in, his door was always open, I expressed my interest on this question of lead. He said he would find his copy of an internal report of the geology concerned and would get back to me when he had found it. As I had only been at EDC for a few weeks, I was not yet aware that Basil Bibby had a continuing ‘game plan' of throwing out intriguing ideas during his lectures to see if any of the postgraduates would ‘bite.' It was his way of identifying those postgraduates who had ‘curiosity' and hence might be suitable for a later career in dental research. Over the years, he had inspired and fostered many academic careers by that approach, including over 100 whose Master of Science degree he personally supervised.

The original EDC building was opened in 1917. Its construction was of the highest quality, fully funded by George Eastman (of Kodak fame), specifically to care for children and to conduct research on the prevention of tooth decay. The building, under George Eastman's insistence, also had dedicated research laboratories. Several staircases led down from the clinic floors to the laboratories and one of the building's characteristics was that all of the staircases had handrails made of solid brass, which were kept highly polished. As postgraduate students, we quickly became aware that we would often encounter Basil Bibby on his way around the building sliding down the handrails, as always in his old brown laboratory coat, like a mischievous schoolboy. This was initially disconcerting for most of us who were more used to directors/professors being dignified individuals of rarified authority.

It was on one such occasion that, as I was passing though some dividing doors into a stairwell, Basil Bibby came whizzing down the brass handrail to meet me. Flourishing a document, he presented it to me as the ‘lead paper you were interested in.' ‘Have a good read,' he said, ‘and drop by sometime and we can talk about it.'

## The research project

The geological paper concerned agricultural land to the west of Rochester where, since the late nineteenth century, there had been reports of ‘some areas of soils being “poisoned” that would not grow crops'. A US geological survey internal report had investigated that problem and recorded heavy metal concentrations as high as high 26,000 parts per million (Zn, Pb, Cd). This I found intriguing and, being curious, stopped by Dr Bibby's office again to discuss it further. Basil agreed it was intriguing and suggested: ‘why don't you drive over there and see what you can find out?'

As I had a free afternoon from clinical duties later that week, I duly drove in my VW Campervan out to the area in question. All this activity, I now know, was part of a planned sequence of events that Basil Bibby devised to see just how interested a postgraduate student might be in a research project. Any instance of curiosity would lead to him inspiring further investigations. At any one time, Basil would have several projects that he accumulated as possible studies for students. There were fellow students in my class at EDC who studied such topics as ‘The effect of spice extracts on acid formation and enamel solubility enamel resistance;' ‘Sugar intake during tooth development;' and ‘Trace element effects on enamel pigmentation and molar morphology in rats,' to name only a few. But there was also topics not related to dental caries, such as ‘The effects of Dilantin on periodontal disease in children.'

My enquiries found that the affected farmland was in a rural area of the county and included several arable farms. Taking a chance and calling at a couple of the farms, I found the famers extremely helpful and willing to walk me down to some acres that were typically affected. In those sites, I saw poor or no growth of crops even though elsewhere within the same field, but higher up a slope, grew a health arable crop, some being maize and some grains (such as wheat or barley). Indeed, those plants that had managed to start growth in an affected area were yellow, weak and stunted. The concentrations of Zn were at 19,000 ppm but more importantly, Pb at 117 ppm, the latter being significantly higher than safe levels. Each farmer expressed an interest and, of course, wanted to know why a dentist would want to study poor agricultural growth. At that stage, I emphasised it was merely a preliminary enquiry.

Reporting back to Dr Bibby he was encouraging (phase two of his encouragement) and then, no doubt as he had planned, posed the question as to how I would think the problem could be investigated and how would I carry out a suitable research study. I had not imagined on starting my postgraduate studies in paediatric dentistry that I would ever be involved in research. I had applied to EDC just to attend the clinical programme and complete the two calendar years leaving with a recognised certificate. As an undergraduate student in dental school, in the late 1950s, student research was not commonly undertaken. However, in my final year at dental school, I had been introduced to research by Professor Dennis Picton and did a tiny project about Nasmyth's membrane.

Despite my limited future career plans or expectations, I thought that this ‘Heavy metals - lead project' was an interesting challenge which would not take up much time, and accordingly, I set out to draw up a scheme to examine the oral status of the children living on the affected farms in Orleans County. Other senior members of the EDC research staff at that time were Drs Fred Losee, Roland Hawes and, a New Zealander on sabbatical, Tom Ludwig. These men were very helpful, and I soon had the outlines of an epidemiological study. Roland Hawes, who was also the chairman of paediatric dentistry, mentioned that part of my paediatric dentistry certificate qualification would require a' minor research project' and the Orleans Lead study would be ideal.

In any epidemiological dental study, there was always the need for a control group to match the test group. To the south of the test county was Genesee County, also mainly farmland, and accordingly that would be suitable. The test community was, therefore, to be a village called Manning and the control community East Oakfield. An explanatory letter was drawn up and a brief consent form devised. Enough copies were made using a Roneo machine and at a time when I had no clinical duties, I spent a day distributing the documents to the farms in both counties. It should be noted that at that time, research ethics review committees did not exist and the paperwork, or protocol as it was later termed, was all that was needed to proceed with research. Accordingly, the proposed project was set up and implemented in just a few days.

## Criminal activity

I was soon to learn that research projects do not always run smoothly and problems that had not been anticipated could arise. A week or so later I was summoned urgently to Dr Bibby's office as there was ‘possibly a slight problem' he needed to advise me about. Attending as soon as possible, Basil told me that the Sheriff's Office of Orleans County, in the county town of Albion, had telephoned to say that they required me immediately to attend their office to discuss ‘a very serious matter' involving criminal activity on my part. Dr Bibby felt I should get out there later that day, find out what the problem was and to sort it out. Very worried about what on earth this could be about, I drove off that very afternoon and found my way to the Albion sheriff's headquarters. Ushered into the Sheriff's Office, I met a large man in resplendent freshly pressed uniform with knife edge creases to both shirt and trousers. On his head, the classical Stetson hat, pistols on his hips as in the cowboy films of my youth. The sheriff was almost identical to Buford T. Justice of *Smokey and the bandit* fame. He introduced himself as Sheriff Swenson and explained that I was at serious risk of being accused of a federal crime carrying a heavy fine if not a prison sentence. My crime was: ‘you've been putting leaflets in US mailboxes, unauthorised and without paying for the postage that was required. That's a federal crime'.

This crime had been reported to the sheriff and he was now required to arrest me for a federal felony, unless of course I had a suitable explanation. Quaking, I started to tell him what I was doing for my student studies. At that point, the Sherrif interrupted me: ‘you're from the Old Country buddy, I can tell from your accent. So, I guess you can be excused for not knowing about our federal laws, right?'.

I immediately assured him that was indeed the case, and I was extremely sorry that I had perpetrated such a heinous crime. With great relief, he accepted my explanation but asked how many letters I had issued. I told him what I thought the number might be, but that I had also ‘posted' some to farms in Genesee County as well. Sheriff Swenson was most helpful and said I had to go to the main Orleans County Post Office in Albion and also to that in Batavia in Genesee County with a document he would give me and pay 15 cents postage on each leaflet. As I had issued 44, leaflets it cost me $6.60.

Fortunately, that ended the problem and I was not arrested and charged with a US felony and had avoided a criminal record, which would have cancelled my student visa to study in the USA immediately and I would been expelled from the country.

## Conducting the research

I needed to get together dental examination instruments, Anglepoise light and the necessary recording sheets. That was not a problem because on the EDC senior staff, recruited by Basil Bibby, were two distinguished dental researchers - Fred Losee and Tom Ludwig. Fred Losee started out as a dental officer in the United States Navy in World War II and eventually had retired from the Navy as a full Captain to spend the rest of his career at EDC in dental caries research. Before settling in Rochester, however, he had been seconded to New Zealand to assist Tom Ludwig, the director of the New Zealand Medical Research Department, Dental Division (see below), as they were investigating geographic differences in the prevalence of dental caries related to water, soils and vegetable composition.

**Fig. 2:**
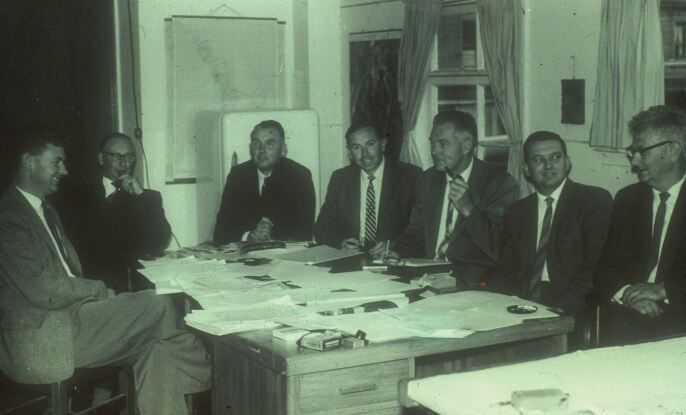
New Zealand Medical Research Council, Dental Unit, 1964. Photograph taken at New Zealand Dental Research Council in 1964. A copy was in the possession of Dr Fred Losee and passed to the author on Dr Losee's death in 1977. Converted by the author to a slide

The photograph in Figure 2 shows the New Zealand Medical Research Council Dental Unit in 1964. Tom Ludwig is seated centre, on his left is Bill Healy (a soil scientist), then Fred Losee third from the right (smoking his pipe as always), plus other members of the research team (P. Cadel; R. Malthus; E. Pearce; J. Darwin). The group, at the time of the photograph, were all involved in the Napier-Hastings dental caries research study, which was initially to demonstrate the effects of water fluoridation. However, the baseline dental caries examinations had shown that the Napier children already had a significantly lower prevalence of dental decay than those in Hastings. That indicated a change in aims of the study as to why that should be. Historical investigations showed that the town of Napier had expanded onto land that had originally been a lagoon raised by an earthquake in 1931. The new land was originally seabed comprising calcified sediments, whereby the soils and hard water supplies for the town were anti-caries. There was an indication that several trace elements were involved.

It was decided that a more extensive study was needed and further instigations led to the award to Fred Losee and Tom Ludwig, now at EDC, of an exceptionally large research grant from the National Institute of Dental Research (USA) to survey dental caries throughout the USA in relation to all types of soils, vegetation, food, trace element composition and tooth decay. Tom Ludwig was seconded from New Zealand to EDC for two years as co-director of the study. Accordingly, all the necessary requirements and materials for the conduct of the study were available.. I therefore had the great honour to be working in a very junior capacity with three renown dental scientists.

As Tom Ludwig was an internationally recognised dental epidemiologist, he was able to teach and calibrate me on the accuracy of diagnosis and recording of dental caries. That was necessary, as any results of my study would need to meet international standards. Tom and Fred had recruited, for the duration of their study, a dental nurse as an assistant and recording scribe and it was arranged that she would assist me.

## Results of the lead study

In total, 42 children and teenagers (3-19 years old) were available for a dental examination. Only those children who had been born and lived in their area all their lives were examined. This was a small sample but the findings, although of extremely limited use, were of interest. The dental caries scores were recorded in a standard way as decayed, missing or filled primary teeth surfaces (dmfs) and the same index for permanent teeth (DMFS). The mean scores were found to be, for the lead area in Manning, as a dmfs of 6.6 and DMFS of 9.7. For comparison, the scores for the control area of East Oakfield were 4.0 and 7.1, respectively. Thus, the indication was for higher dental caries in those children who had lived all their lives in the agricultural area affected by heavy metals, in particular lead. A separate analysis of the number of teeth erupted per child also showed a difference, indicating there was delayed eruption of teeth in children living in the heavy metal area. These findings were in keeping with other studies reported in the literature at that time.

I had now completed phase three of the Bibby scheme of becoming initiated into dental research.

## Developing students' interest in research

As a way of enticing a young dentist into the field of research, it served its purpose. The challenge of identifying an interesting population and setting up the appropriate examinations and analysis of the data, minuscule as it was, sparked a response on my part. Having completed the study, I reported the findings to Basil, Tom and Fred who were encouraging. Basil said he would look at the data in detail and then we could discuss the study further. I thought that now the project was finished, I would continue full-time in clinical dentistry.

After another week had passed, however, a message came from Basil's secretary to drop by his office. As always when I called, he was in his old brown laboratory coat, but accompanied by Fred and Tom, and motioned me to be seated. Complementing me on the results, he felt it was worthy of publication and he would think about which dental journal might be suitable, and that it should be in one of the paediatric dental journals.^3^

However, there was another matter he wished to talk to me about: ‘Dr Ludwig's time at Eastman is running out and there is still more work needed to finish the soils study. As it happens, there is one section in particular remaining which concerns the trace element molybdenum that can produce a toxic pasture for cattle called teart. Doctors Ludwig and Losee were wondering if you might just be interested in doing that small extra study for us?'.

This was unexpected and I was not sure what to say. I mentioned that I was on a full-time course in clinical paediatric dentistry, and I was not at all sure that ‘I would have the time' for another study, which would be larger. I also enquired where the study was needed.

‘Oh,' said Dr Bibby, ‘as it happens, the area of teart pasture occurs in California in the foothills of the Sierra Nevada and a proposed control area is to be in the Napa Valley. I have spoken to Roland Hawes, and he sees no problem with you having some more time off from clinics as you are well ahead and doing very well'.

This completely nonplussed me. What a temptation that was. Travel to California - and at the study's cost - later in the year, including the famous wine area of the Napa Valley! But it was now well into 1967 and would require several weeks away from my family. I took a few days to think about the suggestion and discussed it with my wife, coming to the inevitable conclusion it would be a promising idea and not to be passed up! And so, it came about that I agreed to look in more detail as to what the study required. What had started as curiosity and a simple question following a lecture was now progressing to a larger, more extensive, study. However, it was an opportunity and inspiring to be involved in a much bigger and more important project. Maybe my clinical career plans could be put on hold for a few months more.

It was not long before the gentlemen in question, Basil, Tom, and Fred, intimated that the project could be ideal for a Master of Science degree, ‘if you're interested, of course.'

Not having excelled in grammar school where I struggled to pass the necessary advanced level examinations, further academic study was not welcome. Nevertheless, the opportunity was taken, and I was inspired to set off and develop and carry out what was now called the ‘Molybdenum and dental caries prevalence' project. However, having started the preliminaries for the study, there was another call to see Dr Bibby who was pleased that I had accepted the challenge but, ‘of course, as this is an American Master of Science, you will need to take some advanced academic courses. Fred and I have mulled that over and decided you should do the pharmacology, toxicology and teratology course in the university'.

That announcement certainly was unexpected and a bombshell as I had thought all that was required was the research and a thesis. But not in American universities. Somehow, I managed to complete the academic course in a class of four students, the others all studying for doctorates and all previously qualified in chemistry!

By the late spring of 1968, the ‘Molybdenum study' was finished, the course work passed, and the thesis was being written, when another call came to visit Dr Bibby's office. Yet again, the triumvirate of Basil, Tom and Fred were present. Apprehensively, I sat down not knowing what to expect. Was the study not good enough? Were the results disappointing?

‘We've been very pleased with the Molybdenum project and look forward to your thesis,' said Basil, ‘but another thing has come up. There's a group of people living out in North West Ohio where Fred thinks there is very low levels of decay related to trace elements in water, possibly strontium. If you're not in a hurry to leave Eastman, how about you pop out there to see if Fred's idea is correct?'

‘It's just an idea, of course, but we thought you might be interested.'

Although in early 1968 I had applied to several British dental schools to seek a lectureship in child dental health, nothing had been forthcoming; indeed, there were no vacancies at all. The offer of another interesting research project was, therefore, welcoming and I agreed to undertake the ‘North West Ohio project' which led to another publication.^4^

## Final comments

An expression of curiosity on the part of young students has been the start of many careers, but it needs encouragement by inspirational teachers. Basil Bibby's ability to inspire young postgraduate students was masterful and his challenges became legendry. During the period of Basil's directorship, many careers were developed in teaching, research and clinical care and a significant number came from the British Isles. As shown here, what appeared to be ‘just a small project' became a serious study and from that, a much bigger project, leading to a Master of Science degree. But the outcome was more than just a research project because, as Basil Bibby was so aware, it enabled students, and the author in particular, to start on the ladder to an academic teaching and research career that had never been considered. The role of an inspirational mentor is crucial.
